# Ammonia—Its Relations to Dentistry

**Published:** 1882-03

**Authors:** George Watt

**Affiliations:** Xenia, Ohio


### AMMONIA—ITS RELATIONS TO DENTISTRY.

BY GEORGE WATT, M.D., D.D.S.
Xenia, Ohio.

When the principle of life ceases to hold sway in organized bodies, they begin to undergo a special change. In the language of Divine Inspiration, "They die and return to their dust." To *their* dust, bear in mind; which means that they return to their original elements. To our common progenitor it was said by Him that made him, "Dust thou art, and into dust shalt thou return."

For convenience, the organic kingdom is divided into animal and vegetable sub-kingdoms; but the line of demarkation is not always easily defined. To call that animal which takes oxygen from and gives carbonic acid to the atmosphere, is nearly correct; but as we feel not the need of exactness for present purposes, we shall not hold ourselves under obligation to defend the definition.

The chief bulk of vegetable bodies is made up of carbon, oxygen, and hydrogen; that of animal bodies includes these and also nitrogen. Bodies of which nitrogen is a constituent are often called azotized bodies. When these undergo such decomposition as has been referred to, the process is called putrefaction; while a similar process in non-azotized substances is called fermentation. The exciting cause in each is oxygen. This mighty element lays hold on its luxuries as soon as life has left the body containing them. Carbon and hydrogen are its favorites, its affinity for the latter being the strongest known to chemistry; yet, with this decided advantage, it by no means gets all the hydrogen. Other elements are also ready to seize it, as it is alike ready to lay hold on them; for chemical combinations are always matches of mutual affection. The ordinary state of hydrogen is gaseous, which is not favorable to chemical combinations.

In putrefaction, oxygen takes hydrogen, forming water; it takes carbon, forming carbonic acid; sulphur, forming sulphurous, which rapidly changes to sulphuric acid; it takes phosphorus, forming phosphoric acid. But while all these are going on, other elements are claiming their rights, and some very important combinations take place, as sulphur with hydrogen, phosphorus with the same element; and also nitrogen with hydrogen, forming the alkali, whose name heads our paper. While this is an alkali, the two preceding compounds are acid; and as their formations are simultaneous and in contact, they readily combine. Each has a very offensive odor; but when the alkali combines with either acid, a much more disagreeable one results.

The living body is a constant battlefield between the forces of life and death. The nutritive functions are assimilating and building up, while opposing forces are tearing down and destroying. That is, there is constant warfare between vitality and chemical affinity. Molecules discharge their functional duties in obedience to vital force, and die from the effort, after which they are obedient solely to chemical force. But in building living organs, or tissues, the nutritive functions never violale the laws of chemical action, but each element, or compound radical, in uniting with another element or compound, obeys the laws of chemical equivalents, as strictly as they are obeyed in inorganic chemistry. Vitality is one of the modifying circumstances of affinity, while affinity is one of the supports or sustainers of vitality. The warfare, therefore, as elsewhere in nature, is only apparent, and we may truly say with the poet:

"All nature is but art unknown to thee,
All chance direction which thou canst not see,
All discord harmony not understood,
All partial evil universal good."

From what has been said, it follows that by noting the chemical reactions which follow the death of the individual body, much can be learned as to the results of chemical action on effete matter in the circulating fluid. Hydrogen oxodized in either case yields water, while nitrogen combined with hydrogen, in each case gives ammonia. And as we know that ammonia is abundantly given off from dead bodies rich in nitrogen, such as cadavers of men and beasts, we have a right to infer that the same compound is given off by the combinations which take place in the effete materials within the living body. But, fortunately, we are not left to inference; for common observation, to say nothing of chemical analysis, detects very readily the presence of this compound, at least in the urine, the perspiration and the breath.

Bear in mind that ammonia is always a result of death in azotized, or nitrogenous matter. Its origin in, and its associations with death, are certainly suggestive. These are at least enough to excite suspicion; and wherever and whenever we find it, we should be as inquisitive as where the inhabitants of the city where David dwelt, when the prophet came in search of a candidate for the Kingship, and say to it, "Comest thou peaceably?"

We are not to expect that death will take place in a body consisting in part of nitrogen and hydrogen, without these elements finding each other; and consequently we must regard the formation of ammonia as inevitable. But when no more is formed in the living body than can be taken care of by the acids within the system, not much, if any, harm results; but when such is not the case, the consequences may be serious. And just to the extent that this takes place, the individual dies. And, like other dead or dying bodies, he stinks, even when the ammonia is left free, but much more when it is combined with sulphureted or phosphureted hydrogen. I presume all have noticed a stercoraceous odor about such persons, the odor coming mainly from the breath and the perspiration.

As the effete matter is carried in the blood, the ammonia formed from its decomposition is carried in the same fluid. Its tendency is to dissolve the red corpuscles, and thus the constitution is greatly prostrated, and a very peculiar pallor results, which is not easily described, but which is readily recognized after acquaintance. It is claimed by some pathologists that only in this condition is the constitution liable to erysipelas. This may explain the popularity of tincture of iron as a remedy for this disease, as it has an excess of hydrochloric acid, and ammonium chloride, the salt resulting from its action or combination with ammonia, is highly soluble, which favors its eradication from the system. Whether or not this point in pathology is well taken, the secretions and excretions of the writer were literally loaded with ammonia last February, when he was prostrated with this dread disease.

So much for its deleterious action on the blood. In the breath it may do mischief by its action on the lining membrane of the air-cells, rendering it less fit for the transfer of gases through it; and, to the extent of its volume, it interferes with the reception of oxygen by the blood, as the atmosphere is diluted by its presence. And, like other gases, it obeys the laws of gaseous diffusion, and passes from the air-cells directly into the blood, there to work the mischief already described. But the worst results of breathing an atmosphere laden with ammonia arise from its oxidation, or rather from the oxidation of the elements composing it. According to Liebig, these are always oxidized when brought in contact with oxygen, whether it be nascent or quiescent. As before stated, the oxidation of hydrogen gives water, while the oxidation of nitrogen, whatever may take place as it progresses, finally results in nitric acid. The fact that the resulting water and nitric acid have a strong affinity for each other, Liebig calls a disposing circumstance, which tends to impart increased energy to the process.

A year ago, in this city, the writer and his wife had practical experience in breathing an ammoniacal atmosphere, while it was undergoing oxidation. After this society adjourned last year, we remained over night. A main water pipe had bursted. About 10 P. M., the water was shut off for repairs. This opened the traps of some of the lower water closets. We retired with the transom open in the hall. At midnight we awakened, both unable to speak, scarce able to open the windows and reach the bell signal. Our air passages were clogged with the toughest mucus I ever saw. Our sufferings, for a time, were intense. We had the transom closed, and though the temperature outside was near zero, the windows opened till the atmosphere of the room was changed. No other guests were as much exposed as we, yet, at the breakfast table, the complaints of sore throats and hoarseness were numerous and earnest.

To get a clear idea of the mischief ammonia may do in the mouth we would gain by thinking, for a moment, of the character and composition of the bucal fluids. When normal, the saliva is able to hold all its constituent salts in solution. Its ability to do this depends on the simple fact that it also holds free carbonic acid in solution. Carbonic acid is ordinarily a gas, inodorous, invisible, and nearly tasteless. But when dissolved in water or saliva it is liquid. When liquified thus, or by any other process, it is readily induced to resume its gaseous state. The water of wells and springs in limestone districts is as clear as that found elsewhere, yet it is easy to demonstrate that it holds in solution a quantity of carbonate of lime. You all know that chalk, or marble dust, is not soluble in pure water, but when the water has already dissolved a portion of carbonic acid, it is able to dissolve the chalk or marble. By heating the water the carbonic acid is driven off in its gaseous form, and the carbonate of lime is precipitated, and adheres to any convenient solid, or falls to the bottom as a sediment. All are familiar with the incrustation of boilers and kettles from this source, when "hard water" is boiled in them.

A solution of free carbonic acid is able also to hold in solution the subphosphate of lime, called "bone phosphate," and it is precipitate from its solution just as is carbonate of lime, and under the same circumstances.

It matters not by what process the free carbonic acid is taken from the fluid holding these lime salts in solution, they are precipitated all the same. For example, if the acid is removed by combination with an alkali, the lime salts are precipitated. Dentists are often disappointed to find their patients annoyed by deposits of tartar, even when they are diligently using alkaline dentifrices, which they have prescribed. The explanation is that the alkali combines with and neutralizes the free carbonic acid in the buccal fluids, rendering them totally unable to hold the salts in solution, and they are deposited as tartar.

When the constitution is sound and healthy, when each organ is able to, and does perform its function, the saliva retains its natural amount of free carbonic acid, and hence holds in solution the normal quantity of each lime salt, and no tartar can be deposited. But let the constitution become enfeebled, till the effete matter becomes too abundant for the excretory organs to carry it out of the system, physiology becomes pathology, and organs intended only for secretion, take on excretory work; the salivary glands, or the mucous follicles, or both, give off ammonia, the free carbonic acid in the saliva unites with it, forming carbonate of ammonia, and the saliva is thus rendered totally unable to dissolve the two lime salts, and they with epthelial matter, and deteriorated mucus, are precipitated on the teeth in the form of salivary calculus, or tartar. And now we are able to see whether the disease is constitutional, or merely local. If only local, of course, appropriate topical treatment is all that is necessary to its successful management; and there is justification for those who claim to know all about the disease, yet rely solely on a thorough scraping for its cure.

A fertile source of ammonia within the mouth may yet be noticed. The organic matter entangled in the lime salts, and thus constituting a portion of tartar, decomposes, yielding the ordinary products of putrefaction, and consequently ammonia. This fact alone calls for the thorough scraping or cleansing universally recommended and practiced by all who are conscientious and industrious. Other reasons call for the same treatment, but the one reason is sufficient. To get rid of the ammonical odors of our stables, we scrape and shovel out the manure; but if the horse is declining, we scarcely rest content with this alone. The physician who in treating zymotic disease—typhoid for example—would be ridiculed if he should rely on the removal of the excrements as the only means of cure; and he would be rightfully censured if he should allow them to remain in the sick room during treatment.

It would appear, then, that we have no more right to regard and treat this morbid condition as local, than if it were erysipelas, scurvy, scrofula, or consumption. Nor will it do to rely wholly on either local or general treatment, but a legitimate use of both is the proper course.

As to the topical treatment, it has already been intimated that the most thorough removal of all deposits about the necks of the teeth, and especially between the free margins of the gums and the teeth, is necessary. All tartar must be removed, and the teeth must be polished. And if parts of the margins of the gums have so far lost their vitality that normal, healthy circulation cannot be re-established in them, they should also be removed. And the same teaching applies with equal force to the borders of the sockets, if they too have lost vitality. There is not much danger of overdoing in this direction. Some cases require many sittings, with patience and perseverance on the part of both patient and practitioner. It is not in accordance with the plan of this paper to suggest instruments, or describe manipulations. Let the instruments be the best possible in quality and adaptability, while judgment and skill must guide the operator and control his manipulations.

The mouth having been thoroughly cleansed, it follows to make effort to restore the lost tone to the vessels and fibers of the tissues involved. Tonics and astringents, with friction, may be used, the friction being the most important of these suggestions. Almost any wash may be suggested that necessitates friction in its application Friction with the tooth-brush is not usually sufficient. The morbid blood is not thereby forced out of the loaded vessels. Firm and persevering friction with the finger over all accessible portions of the gums should be resorted to at least three times a day. The superficial vessels are thus emptied, and the expelled blood does not directly return to them, but that which is fresher and more nutritive takes its place. This already detailed, or other topical treatment to accomplish the same results, is by many regarded as quite sufficient; and with this much done, in many cases nature will be aroused to accomplish the rest. But in aggravated cases, the patient will soon be found as badly off as before, if nothing more is done. If the constitution is still in such state of deterioration that the mucous follicles of the air passages and the salivary glands are called on to aid in the elimination of ammonia, the saliva cannot hold the lime salts in solution, and tartar must be deposited. The indication is, therefore, to get rid of the surplus ammonia. But how?

Bearing in mind that ammonia is a result of the putrefaction of dead or effete nitrogenous tissue, we must obviate the tendency to death. The weakened organs of the constitution must be restored to their normal power. The excretory organs, if torpid, must be aroused to do their whole duty, so that those whose duty is secretion shall not be called on to excrete. And, fortunately, in many cases the remedies needed as tonics and alteratives for weakened and depraved organs, are such as at the same time neutralize the excess of the objectionable alkali. It is well known that the mineral acids are popular remedies in indigestion where the fault is with the liver or stomach, or even with both. While they stimulate the coats of the stomach to a proper secretion of mucus, and the normal supply of the gastric juice, they also stimulate the liver to an increased secretion of bile, and thus relieve obstruction in the portal circulation, and the consequent congestion of the abdominal and pelvic organs, and at the same time they neutralize the excess of ammonia in the circulation (thus saving the red corpuscles from solution), forming with it highly soluble neutral salts, nearly or quite harmless in the blood, yet easily eliminated therefrom by the emunctories. For such purpose the aromatic sulphuric acid, called elixir vitriol, is often administered. It may be given in doses of ten to thirty drops three or four times a day, diluted with water. Or nitro-muriatic acid, called *Aqua Regia*, may be used for the same purpose and in the same way, but in doses of three or four drops. Better results, however, may be obtained from *aqua regia* by using it as a bath. Officinal *aqua regia* is prepared by mixing one part of nitric with two parts, by measure, of hydro-chloric acid; and in preparing the bath, four to six ounces of it may be added to three gallons of water. The feet may be immersed in the bath, or the liquid may be applied to the surface of the body by a sponge.

If nitric acid is preferred, the officinal dilute acid may be used as directed for elixir vitriol; and the same direction will answer for hydro-chloric acid, but when this is needed it is common to prescribe tincture of iron, which has in it an axcess of the acid; and the iron aids in restoring the red corpuscles to replace those destroyed by the ammonia. This may be given three or four times a day, in doses of ten to twenty drops.

But often relief may be obtained by a free use of vegetable acids. Acetic acid is universally used, and often in spite of strong prejudice. The growing girl drinks vinegar on the sly, and steals pickles and eats them between meals, almost without chewing, and if she suffers from indigestion her case is cited in proof that pickles are not wholesome food. Some times we find the prejudice against vinegar so strong, on the part of parents and proprietors of boarding schools, that we have to take advantage of a Latin prescription in order to have it used by the patient. Perhaps no organic acid meets the demand better than acetic, in the class of cases under consideration. Fortunately it is cheap and abundant. But for a patient in fashionable life, it is sometimes better to prescribe citric acid in the shape of lemons, or lemonade. The additional cost sometimes insures attention, and the taste is, perhaps more pleasant. These acids may be used almost at will by the patient, but in general should be used only with the regular meals, or with lunch at a definite hour. The stomach should be taxed with nothing but water between meals. This liquid needs no digestion, but passes unchanged into the blood. Bearing this in mind will aid in recalling the principle that should guide in dietary affairs, that anything requiring digestion as a prelude to assimilation, should be taken only at regular meals. We are thus minute because proper nutrition is necessary to the prevention or cure of the morbid state we have been discussing.

Acid fruits often afford relief, and of these the grape is, perhaps, best of all. Strawberries are excellent. Apples, currants, cherries answer a good purpose, as in many cases they not only neutralize an excess of ammonia already formed, but they prevent excessive formations of it, by imparting a healthy tone to the digestive apparatus, and thus building up the constitution to a state of vigor incompatible with the excessive formations of the objectionable alkali.

When an aggravated case of disease resulting in the abundant formation of ammonia presents itself, the practitioner should make up his mind for persevering attention. Of course filling teeth should not be thought of till the mouth is clean, and the gums are healthy and solid. A number of visits will be needed in most cases, and the patient should be impressed with the idea that the restoration of the mouth to health and decency is far more important than filling cavities of decay. Let them know that after the periods of childhood and youth are past, many more teeth are lost by this ammoniacal degeneration, than are lost by decay. And when the public mind is educated up to understand that the most important and the most difficult part of our practice is to combat disease, they will better appreciate dental surgery, and will not estimate the value of our services by comparing the bulk of metal used in filling a tooth with that used by the blacksmith in shoeing horses. They will come to believe that mind is above manipulation.

